# In reply to: “Letter to the editor in response to the article:‘Squamous cell carcinoma arising in chronically damaged skin (Marjolijn’s Ulcer)’”

**DOI:** 10.1093/oncolo/oyaf161

**Published:** 2025-06-26

**Authors:** Mor Miodovnik, Yardenna Dolev, Roni Buchen, Miriam Rivka Brezis, Alla Nikolaevski-Berlin, Inbar Finkel, Ido Wolf, Inna Ospovat, Orit Gutfeld, Yasmin Leshem

**Affiliations:** Oncology Division, Tel Aviv Sourasky Medical Center, Tel Aviv, 6423906, Israel; Oncology Division, Tel Aviv Sourasky Medical Center, Tel Aviv, 6423906, Israel; Sackler Faculty of Medicine, Tel Aviv University, Tel Aviv, 69978, Israel; Oncology Division, Tel Aviv Sourasky Medical Center, Tel Aviv, 6423906, Israel; Oncology Division, Tel Aviv Sourasky Medical Center, Tel Aviv, 6423906, Israel; Oncology Division, Tel Aviv Sourasky Medical Center, Tel Aviv, 6423906, Israel; Oncology Division, Tel Aviv Sourasky Medical Center, Tel Aviv, 6423906, Israel; Sackler Faculty of Medicine, Tel Aviv University, Tel Aviv, 69978, Israel; Oncology Division, Tel Aviv Sourasky Medical Center, Tel Aviv, 6423906, Israel; Oncology Division, Tel Aviv Sourasky Medical Center, Tel Aviv, 6423906, Israel; Oncology Division, Tel Aviv Sourasky Medical Center, Tel Aviv, 6423906, Israel; Department of Systems Immunology, Weizmann Institute of Science, Rehovot, 7610001, Israel

We appreciate Ackerman et al. interest in our work.^1^ In their letter, they point to a potential role of local iron overload in chronic ulcers resulting from venous insufficiency and their contribution to skin damage eventually leading to malignant transformation. Dr Ackerman highlights the hypothesis that iron accumulation within chronic venous ulcers may contribute to oxidative stress, genetic instability, and an immunosuppressive microenvironment, potentially influencing the progression and treatment response of ulcer-associated squamous cell carcinoma (SCC-MU).^2,3^ While our study did not directly assess iron levels in these tumors, we agree that chronic inflammation and microenvironmental factors including iron overload are likely to play significant roles in the pathogenesis and therapeutic resistance of SCC-MU. However, it is important to note that the majority of Marjolin ulcers are not associated with venous insufficiency. Other well-recognized predisposing conditions for MU include burn scars, osteomyelitis, pressure sores, and surgical scars.^4^ Furthermore, even in cases of venous insufficiency, a direct causal relationship between iron overload and skin cancer formation has not been firmly established. While iron has been implicated as a potential carcinogen, the evidence remains inconclusive, and further research is warranted to clarify this association.^5^

In our article, we proposed that the pathogenesis of nonulcer-type cutaneous squamous cell carcinoma (cSCC) is driven predominantly by sun exposure, resulting in a high mutational burden. In contrast, we hypothesize that inflammation-driven cSCC, such as Marjolin’s ulcers, may carry a lower mutational load. This could explain differences in immunogenicity and response to immunotherapy between these subtypes. The complex interplay between chronic inflammation, tumor mutational burden, and immune evasion remains a compelling area for further investigation.
